# A Cross-Sectional Investigation for Verification of Globalization of Falsified Medicines in Cambodia, Indicated by Tablets of Sildenafil Citrate

**DOI:** 10.3390/pharmacy7030111

**Published:** 2019-08-09

**Authors:** Naoko Yoshida, Miku Yuasa, Tey Sovannarith, Eav Dararth, Tep Keila, Heng Bun Kiet, Hirohito Tsuboi, Tsuyoshi Tanimoto, Kazuko Kimura

**Affiliations:** 1 Clinical Pharmacy and Healthcare Science, Faculty of Pharmacy, Institute of Medical, Pharmaceutical and Health Sciences, Kanazawa University, Kakuma-Machi, Kanazawa 920–1192, Japan; 2 National Health Product Quality Control Center, Ministry of Health, 151–153 Kampuchea Krom St., Khan 7 Makara, Phnom Penh 12250, Cambodia; 3 Department of Drugs and Food, Ministry of Health, 151–153 Kampuchea Krom St., Khan 7 Makara 12250, Phnom Penh, Cambodia; 4 Osaka Office, Pharmaceutical and Medical Device Regulatory Science Society of Japan, Sawanotsuru building 8th Floor, 2-1-2 Hiranocho, Chuo-Ku, Osaka 541-0046, Japan; 5 Medi-Quality Security Institute, Graduate School of Medical Sciences, Kanazawa University, Kakuma-Machi, Kanazawa 920-1192, Japan

**Keywords:** falsified medicines, sildenafil citrate, globalization

## Abstract

Medicine falsification is a global issue. Viagra, an erectile dysfunction therapeutic (EDT) medicine consisting primarily of sildenafil citrate, is the most commonly falsified medicine worldwide. Recently falsified EDTs have been reported multiple times in developing countries. The globalization of falsified EDTs has become a concern. In the present study, we selected sildenafil citrate tablets as an indicator and examined samples from a developing country, Cambodia, to investigate the availability of falsified sildenafil tablets in Cambodia and verify the current globalization status of falsified medicines from the standpoint of a developing country. Six samples of the originator Viagra, and 68 samples of generic sildenafil products were purchased from private drug outlets and wholesalers in Phnom Penh, Svay Rieng, and Battambang. The samples’ manufacturers were contacted to authenticate the samples. The quantities and dissolution rates of active ingredients were measured by a high-performance liquid chromatography system with photodiode array. Five generic samples were strongly suspected to be falsified medicines because of their extremely low quality; however, there was little distribution and no falsified medicine alleged to be produced by the originator of Viagra, which charges high prices. That finding indicates that falsification reflects local economic circumstances.

## 1. Introduction

Falsified medicines are those that deliberately and fraudulently misrepresent their identity, composition, or source, and they cause damage to human health and fund organized crime; hence, they should be eliminated from pharmaceutical distribution networks [1]. The Pharmaceutical Security Institute reports the number of incidents involving falsified medicines yearly, which is increasing [2]. Almost 1400 substandard and falsified (SF) medical products have been reported to the World Health Organization (WHO) Global Surveillance and Monitoring System since July 2013 to February 2017 [3].

Health hazards caused by falsified medicines have not been limited by region. The prevalence of falsified medicines in developed countries has expanded exponentially since Viagra, an erectile dysfunction therapeutic (EDT) medicine containing sildenafil citrate of phosphodiesterase type 5 inhibitor, marketed by Pfizer Inc. (New York, NY, USA), in 1998 [4], surpassed the falsified pharmaceutical market of antimalarials and antibiotics as the most commonly reported ones on a global basis [1]. There are distinctions between regions, and falsified EDTs were reported in developing countries as the most common fake drugs. In Thailand: 96,432 tablets, consisting of 83,260, 12,148, and 1024 tablets of Viagra, Cialis, and Levitra, respectively, were seized by The Thai Food and Drugs Administration in 2008 [5,6]. Thus, EDTs (i.e., lifestyle and not lifesaving medicines) have become a target for drug falsification and should be verified to prevent health hazards caused by falsified medicines.

Verification of the globalization of falsified medicines is necessary to identify and control the spread of falsified medicines. The overall growth in falsified medicines is considered to be partially attributable to sildenafil tablets. Accordingly, EDTs are representative of the overall situation with falsified medicines. There have been reports on falsified Viagra in developed countries’ pharmaceutical marketplaces, but little information remains available from developing countries. Therefore, an investigation of falsified medicines in developing countries is necessary. In the present study, we selected sildenafil citrate tablets as an indicator; the study was performed with samples from a developing country, Cambodia, to generate real and reliable information about its situation of drug falsification.

The aims of this study were to investigate the current distribution situation of falsified sildenafil tablets and their quality in Cambodia, and verify the current globalization status of falsified medicines from the standpoint of a developing country to facilitate prevention of the global spread of falsified medicines.

## 2. Materials and Methods

### 2.1. Materials

Viagra (100-mg tablets), which was used as a reference, and a reference standard (RS) of sildenafil citrate, were generously donated by Pfizer Japan, Inc. (Tokyo, Japan). Sulfadoxine was purchased from Wako Pure Chemical Industries, Ltd. (Osaka, Japan). High-performance liquid chromatography (HPLC)-grade acetonitrile was purchased from Nacalai Tesque, Inc. (Kyoto, Japan). All other chemicals were commercially available and of analytical grade.

### 2.2. Sampling

On 9–16 June 2010 and 8–17 June 2011, a group of Japanese researchers and Cambodian collaborators purchased sildenafil while speaking Cambodian in different markets located in the capitol (Phnom Penh in 2010 and 2011) and two provinces (Svay Rieng in 2010 and Battambang in 2011). Svay Rieng is one of the poorest provinces in Cambodia, and it lies on the border with Vietnam. Battambang is a large province located along the border area with Thailand. The convenience sampling that was implemented in this study was the only method that was feasible during the study period.

The sildenafil samples were collected from private drug outlets and wholesalers. All sildenafil samples collected in 2010 and up to 30 Viagra and generic samples collected in 2011 were purchased from private drug outlets. In Phnom Penh, the outlets visited were selected randomly using a list of drug outlets. In Svay Rieng and Battambang, all of the outlets found by researchers were visited.

### 2.3. Observation

For each sample, the packaging condition, package insert, labelled amount of active ingredient per tablet, and package label information were carefully checked after collection.

### 2.4. Authenticity

The authenticity and registration status of each sample were investigated by modified methodology of World Health Organization [7,8,9,10,11]. To determine whether or not the collected samples were genuine, their manufacturers were contacted to verify their authenticity, and we also analyzed their legality as per their regulatory authorities.

### 2.5. Quality

The quality tests were performed according to a previously described method [12]. Quantity tests (*n* = 71) and dissolution tests (*n* = 54) were performed. Three or six tablets from each sample were tested with a dissolution time of 15 min. The acceptable range was set as 90–110% of the labeled quantity of overall sildenafil, with no unit permitted <75% or >125% of the labeled amount. For evaluation of dissolution, the acceptable average dissolution rate for three or six units was ≥75% of the target rate, with no unit allowed a dissolution rate of <50%.

### 2.6. Assay

The sildenafil citrate assay was performed with reference to a previously described method [13]. Determination of sildenafil was conducted on a column of Shim-pack VP-ODS (4.6 mm × 150 mm, 5 μm, Shimadzu GLC Ltd., Tokyo, Japan) using an HPLC system with photodiode array. The mobile phase was a mixture of 0.05-M phosphate buffer (pH 3.0) and acetonitrile (73:27, *v*/*v*). The flow rate was 1.0 mL/min, and the detection wavelength was set at 290 nm. Ultraviolet spectra 200–400 nm were monitored. The internal standard method was used and sulfadoxine was added as the internal standard (IS) in our assay. A calibration curve was created using the ratio of the peak areas of the RS and IS, and then the amount of sildenafil in each tablet was quantified from the calibration curve.

### 2.7. Sample Preparation

For tablet samples, we weighed 10 mg of milled tablet, which was sonicated with an adequate amount of mobile phase for 15 min and diluted to 20 mL in a volumetric flask. We transferred 1 mL of that diluent to another tube, to which we added 1 mL of mobile phase and another 0.5 mL of IS solution (0.1 mg/mL in mobile phase). The diluent was passed through a membrane filter (pore size: 0.20 μm, Millex^®^-LG, Merck Millipore, Billerica, MA, USA) and analyzed.

### 2.8. Statistical Analysis

Fisher’s exact test was used for quality-related factors. Student’s t-tests were used for relationships with prices and quality. *p*-values of <0.05 were considered statistically significant.

### 2.9. Ethical Approval

The implementation of this sampling was approved by the Cambodian government. This study was ineligible for ethical review by Kanazawa University.

## 3. Results

A total of 209 drug outlets (including 14 wholesalers) were visited, and 74 samples of sildenafil tablets were collected from 54 drug outlets (including 7 wholesalers) in the study area (Table 1). The originator, Viagra, was sold in only five outlets in Phnom Penh (Table 1); six samples of Viagra manufactured in Australia (8.1%) and 68 samples in 10 generic medicine products manufactured in India (91.9%) were purchased (Table 2). The price of Viagra (12.3 ± 2.7 dollars/tablet) was twentyfold higher than that of generics (0.51 ± 0.18 dollars/tablet), a significant difference (Student’s *t*-test, *p* < 0.01). Of the samples, 43 (58.1%) were collected from Phnom Penh, including six samples of Viagra; and 31 (41.9%) were collected from the provinces, six (8.1%) from Svay Rieng, including two samples from illegal outlets, and 25 (33.8%) from Battambang (Table 3).

An Australian manufacturer, six Indian manufacturers, and an Australian regulatory authority replied to the authenticity investigation of samples collected in 2010. For samples collected in 2011, an Australian manufacturer and an Indian manufacturer had replied. Out of the 74 samples, replies from manufacturers were received, regarding 44 confirming them as genuine (Table 4). Among of the 11 manufacturers, the manufacturer of Viagra confirmed the legitimacy of its product, and the identity of that manufacturer was confirmed by the regulatory authority in its country of manufacture. All manufacturers who replied had approved Good Manufacturing Practices. Out of the 74 samples, 73 (98.6%) were registered by the Department of Drugs and Food in Cambodia, but a sample of a generic one (1.4%) was not registered.

**Figure 1:**
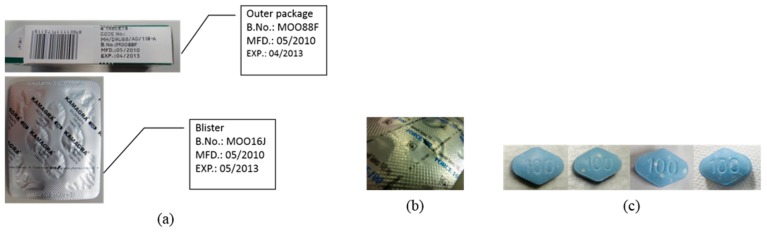
Abnormal points found in the observation test. (**a**) The batch number and expiry date were different from those on the outer package and blister pack in five samples of product F. (**b**) There was a hole in the blister packin a sample of product D. (**c**) The appearances of the film coating were different across all tablets in five samples of product J.

The observation test showed that 11 samples were abnormal (Figure 1). In five samples of product F, the batch number and expiry date differed between the outer box and blister pack (Figure 1a). In a sample of product D, there was a hole in the blister (Figure 1b). In five samples of product J, there was unevenness in the film coating of each tablet (Figure 1c).

**Figure 2:**
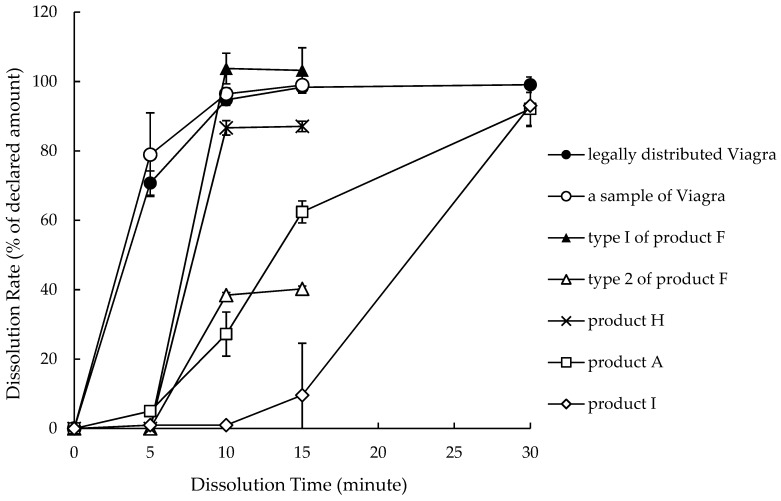
Time course of dissolution for each sample.

For the quality tests, quantity and dissolution tests were performed on 71 and 54 samples; the results indicated that 9 (12.7%) and 8 (14.8%) samples were unacceptable, respectively. Collectively, 12 samples were judged as poor-quality medicine, and all of them were generics. Poor-quality medicine was not found in 42 samples, including all those of Viagra. Either one or no tests were performed on another 20 samples, as the insufficient number of samples prevented quality verification. The detailed results of the quality tests on poor-quality medicines are shown in Table 5. In the quantity test, the mean of quantity of five samples of product F was <50% of the labeled amount. Four samples of product H contained 80–90% of the regulation quantity of active ingredient. Furthermore, we compared the calculated amount of excipients in the quality tested tablets between the standard quantity (i.e., without failure) and substandard samples in the same dosage form of products (Table 6). For products A and product F, the excipient content levels in substandard samples were significantly higher than those in standard samples. In product I, there was no significant difference in excipient content between standard and substandard samples. The dissolution test found that the mean dissolution rates of a sample of product A and two samples of product I were <75% of the declared rate, but the quantity of those samples was adequate. The dissolution profiles of some samples, including poor-quality ones, are shown in Figure 2. Although samples of Viagra dissolved promptly, the dissolution progress of poor-quality samples was delayed: The dissolution rate of two samples (product A and I) only equalized with that of Viagra after 30 min. The dissolution rate of the poor-quality sample of product F plateaued around 40% after 10 min, meaning that its dissolution reached 100% of the actual quantity at 10 min. Active ingredients other than sildenafil were not detected by this analytical method using HPLC with a photodiode array.

We divided the 14 samples of product F manufactured in India into two major types (i.e., types I and II) according to the observation and quality tests. Of the 14 samples of product F, no abnormal points were detected among nine samples, and they contained an adequate amount of sildenafil (99.7 ± 4.0%; type I). The other five samples of product F had different batch numbers and expiry dates between the outer package and blister pack, a low quantity of active ingredient (43.8 ± 4.6%), and no hologram, despite type I having a hologram on the outer package (type II). From these findings, type II was highly suspected of being falsified medicine.

To determine the characteristics of low-quality medicine, the association between sample quality and each factor was examined. No significant associations between quality and type of shop, area, or price were found, and quality might be related to whether or not answers could be obtained during the authenticity investigation. No falsified medicines were detected in samples for which replies were received, and we did not obtain an answer from the manufacturer of product F. Quality might also be related to abnormal observations (Table 7): The poor-quality medicines showed a significantly higher incidence of abnormal observations (5/12 samples) than did samples with adequate quality (4/42 samples; Fisher’s exact test, *p* < 0.05).

## 4. Discussion

Only a small amount of originator Viagra was available in Cambodia during the purchasing (Table 1), and falsified Viagra was not found in this study; however, falsified Viagra has been found several times previously [14,15]. A possible reason for Viagra’s low distribution in Cambodia is its high price (Table 2). The price of generic sildenafil products was less than Viagra, and generic sildenafil products were sold in some outlets in Cambodia (Table 2). Nevertheless, the amount of sildenafil products in circulation might not be large. However, we found that some samples of product F, collected in this study were strongly suspected of being falsified medicines. Of the 14 samples of product F collected for this study, 5 contained less than 50% of the declared amount of active ingredient, and their packages and tablets had several suspicious characteristics, such as no hologram or different information in outer package and blister pack (Table 5, Figure 1 and Figure 2); hence, these samples were strongly suspected of being falsified medicines. This finding suggested that the target medicine for falsification might depend on economic development level, such as income and standard of living. It is commonly accepted that the most frequently falsified medicines in wealthy countries are expensive lifestyle medicines, such as EDTs and diet pills; however, in developing countries, they have included lifesaving medicines, such as anti-infective agents [16]. The existence of numerous falsified medicines, such as antimalarials and antibiotics, and the health hazards caused by them in Cambodia have been previously reported [17,18,19,20]. In other developing countries, fatal cases caused by falsified lifesaving medicines, such as falsified anti-infective agents, meningitis vaccines, and antipyretics, have been reported [19,21,22,23,24,25,26,27]. This study’s results supported the common view and provide a reassessment of falsified medicines’ status in the world.

The manufacturer of product F never replied to the questionnaire for the authenticity investigation. In response to the results of this study, the Cambodian registration of product F was withdrawn by the Department of Drugs and Food in 2011. Additionally, some substandard medicines were found. Among the three generic sildenafil samples, the correct quantity of active ingredient was contained, but their dissolution rates were below the acceptable range. Substandard medicines are a huge problem in developing countries’ pharmaceutical marketplaces, and quality control improvements at manufacturing sites and throughout distribution channels are urgently necessary. In response to this situation, a range of measures are being implemented by the Ministry of Health of Cambodia against these poor-quality medicines to improve the quality of medicine, and this could improve the situation [12].

Authentication was not completed for nearly half of the samples collected in this study, because some manufacturers and regulatory authorities did not reply, despite repeated reminders (Table 4). The cooperation of the manufacturers and regulatory authorities is essential to survey quality, and a higher response rate could improve medicine quality and contribute to quality management. However, some generic sildenafil samples were confirmed as genuine, even though they were assessed as low-quality medicines. Furthermore, abnormal observations, such as mismatch of the product numbers between the outer package and blister pack, the existence of a torn blister pack, and uneven coloration, were found more frequently in the poor-quality medicines (Table 7). These findings indicated that compliance with Good Manufacturing Practices might not be enough; product quality-checking and verification systems in manufacturing processes should be enhanced.

Many generic medicines manufactured in India were collected for this study, and some of them may be suspicious of falsified medicines. Even if all of them were genuine, problems were found in the quality tests. To prevent health hazards, the quality management of medicine distribution should be enhanced in developing countries, because pharmaceutical distribution has become a more global enterprise. The maintenance of medicine quality levels in each country could extricate SF medicines from pharmaceutical distribution pathways. It is difficult for consumers to distinguish medicines of poor quality; to prevent the hazards threatened by SF medicines purchased in markets, it is essential to investigate the circumstances of SF medicine distribution, select appropriate medicines to reflect the distribution situation in target area, and monitor falsified medicines. Furthermore, it is necessary to develop strategies to inform consumers about the risks of falsified medicines; that is why we expect to continue our studies of medicine quality control and why global studies are needed. In this manner, the global status of medicines could be monitored.

In this study, we analyzed the content level and dissolution of the medicines’ active ingredient and found that generic sildenafil samples distributed in Cambodia contained underdoses of the active ingredient. The lowering of the quantity of active ingredients in the medicines constitutes serious deception and may be a criminal act. In the study of falsified medicines, it might be necessary to consider the percentage of active ingredient in the whole tablet mass, and the amount of excipients contained in the tablet, to make comparisons with the genuine composition. However, making such comparisons requires obtaining a genuine, properly stored product from the manufacturer. In this study, substandard tablets whose falsification was strongly suspected had higher excipient content levels than other tablets had (Table 6). It is also important to determine the composition of the excipients in the tablets because falsified medicines may contain harmful substances, although this was not clarified in this study because of insufficient resources.

Although its sampling was done about a decade ago, this report could provide important information to track the status of medicine falsification and the distribution of falsified medicine. It is desirable to investigate this situation’s current status further and observe any changes. The limitations of this study include selection bias. This study might not represent actual conditions in Cambodia accurately because the samples were collected in limited Cambodian outlets. Some outlets for sampling in Phnom Penh were selected randomly by using a list of outlets; however, outside Phnom Penh, they were visited on sight. Although Phnom Penh and Battambang are comparatively developed areas, a low distribution of originator Viagra was found. In more rural areas, its distribution might be even lower. However, circumstances in developing countries may change according to lifestyle. To describe the situation of pharmaceutical quality accurately, its common distribution status in the target country should be considered.

## 5. Conclusions

Viagra, an expensive lifestyle medicine, was not found to be widely distributed in Cambodia while cheaper generics are more prevalent. In this study, no falsified Viagra was found, but poor-quality medicines, which were strongly suspected as falsified medicines, were found among generics. The existence of poor-quality medicines could remain a continuing threat to patients, and the distribution of SF medicines could reflect the regional situation.

## Figures and Tables

Dissolution profiles of legally distributed Viagra (closed circles), a sample of Viagra (open circles), samples of product A (open squares), type I of product F with no abnormal point (closed triangles), type II of product F with poor quality (open triangles), product H (“x” marks), and product I (open diamonds) as mean values with standard deviations (*n* = 3).

**Table 1:** Numbers of sales outlets.

Area	Shop	Year	Visited	Product Sold	
				Viagra	Generic
Phnom Penh	outlet	2010 2011	68	1	24
			57	2	0
	wholesaler	2010 2011	14	1	5
			8	1	0
Battambang	outlet	2011	32	0	14
Svay Rieng	outlet	2010	29	0	6
Total			209	5	49

**Table 2:** Outline of samples collected.

Category	Product Code	Manufacturing Country	Number of Samples (*n* = 74)	Price per Tablet (US $, Mean ± SD)
Originator	Viagra	Australia	6	
subtotal			6	12.3 ± 2.7
Generic	A	India	2	
	B	India	4	
	C	India	3	
	D	India	7	
	E	India	1	
	F	India	14	
	G	India	1	
	H	India	6	
	I	India	9	
	J	India	21	
subtotal			68	0.51 ± 0.18

**Table 3:** Outline of sampling.

Items		Collected Samples	
		Number	%
Sampling area	Phnom Penh Battambang Svay Rieng	43	58.1
		25	33.8
		6	8.1
Shop category	Legal (Pharmacy) (Depot A) (Depot B) Illegal Wholesaler	63	85.1
		(36)	(48.6)
		(7)	(9.5)
		(20)	(27.0)
		2	2.7
		9	12.2
Strength	100 mg	64	86.5
	50 mg	10	13.5

**Table 4:** Results of authenticity investigation.

Items			Originator (Viagra)	Generic	Total
Manufacturers’ reply (*n* = 74)					
Sample authentication		Genuine/replied Other	6/6	38	44
			0	30	30
Regulatory authorities’ reply (*n* = 11)					
Product legitimacy		Legitimate/replied Other	1/1	0	1
			0	10	10
Manufacturer legitimacy		Legitimate/replied Other	1/1	0	1
			0	10	10
Registration number (*n* = 74)					
		Existed Nonexistent	6	67	73
			0	1	1
Registration status (*n* = 74)					
		Registered Other	6	67	73
			0	1	1

**Table 5:** Results of quality test of poor-quality samples.

Sample Code	Product Code	Quality (Mean ± SD)		Answer in the Authenticity Investigation
		Quantity (%)	Dissolution (%)	
A003/CB11	A	95.8 ± 2.4	63.6 ± 5.1	Not obtained
A076/CB10	F	37.3 ± 1.7	39.5 ± 1.0	Not obtained
A055/CB10	F	39.3 ± 1.0	40.2 ± 0.9	Not obtained
PP002/CB10	F	49.2 ± 3.8	47.4 ± 0.4	Not obtained
A066/CB10	F	46.0 ± 2.0	48.3 ± 1.4	Not obtained
A120/CB10	F	47.1 ± 1.2	49.6 ± 1.8	Not obtained
A100/CB10	H	86.8 ± 4.6	93.9 ± 0.5	Not obtained
A119/CB10	H	80.4 ± 1.7	87.1 ± 1.5	Not obtained
B021/CB10	H	81.6 ± 1.3	86.6 ± 2.4	Not obtained
A138/CB10	H	88.3 ± 0.3	91.0 ± 0.8	Not obtained
PAB002/CB10	I	101.1 ± 4.6	6.4 ± 10.4	Not obtained
A043/CB11	I	95.2 ± 8.3	56.9 ± 48.1	Not obtained

**Table 6:** Comparison of excipient contents between standard and substandard samples.

Product Code	Dosage Form (mg/Tablet)	Quality	Number of Tested Tablets	Mean ± SD (%)	*t*-Test	*P* Value
Viagra	50	Standard Substandard	2	84.40 ± 0.352 * No value	Not tested	No value
			0			
	100	Standard Substandard	12	84.20 ± 0.257 No value	Not tested	No value
			0			
A	100	Standard Substandard	6	83.48 ± 0.454 84.02 ± 0.071	Student	0.016
			6			
B	100	Standard Substandard	18	83.84 ± 0.351 No value	Not tested	No value
			0			
C	100	Standard Substandard	12	82.54 ± 0.324 No value	Not tested	No value
			0			
D	100	Standard Substandard	36	82.91 ± 0.403 No value	Not tested	No value
			0			
E	100	Standard Substandard	3	82.32 ± 0.203 No value	Not tested	No value
			0			
F	100	Standard Substandard	33	76.87 ± 7.586 93.02 ± 0.626	Welch	<0.001
			24			
G	50	Standard Substandard	3	84.19 ± 0.422 No value	Not tested	No value
			0			
H	50	Standard Substandard	3	87.58 ± 0.572 No value	Not tested	No value
			0			
	100	Standard Substandard	12	75.34 ± 2.188 No value	Not tested	No value
			0			
I	50	Standard Substandard	33	82.20 ± 0.696 No value	Not tested	No value
			0			
J	100	Standard Substandard	6	67.47 ± 0.773 68.94 ± 2.086	Student	0.119
			12			
	100	Standard Substandard	105	81.97 ± 0.547 No value	Not tested	No value
			0			

* Statistically meaningless because of insufficient number of tested tablets.

**Table 7:** Comparison between observation and quality.

		Quality	
		Number of Adequate Samples	Number of Poor Samples
Observation	Number of normal samples	38	7
	Number of abnormal samples	4	5
